# Effect of Cold-Sprayed Zinc Coating and Heat Treatment on the Microstructure and Corrosion Behavior of 30MnB5 Hot-Stamped Steel

**DOI:** 10.3390/ma18215032

**Published:** 2025-11-05

**Authors:** Hyunbin Nam, Minseok Seo, Cheolho Park

**Affiliations:** Department of Welding & Joining Science Engineering, Chosun University, Gwangju 61452, Republic of Korea; hbnam12@chosun.ac.kr (H.N.); minseok0724@chosun.ac.kr (M.S.)

**Keywords:** hot-stamping process, Al–Si coating, zinc coating, sacrificial anode, microstructure, corrosion behavior

## Abstract

This study investigated the microstructure and corrosion behavior of 30MnB5 hot-stamped steel after applying a zinc coating using the cold-spraying method followed by heat treatment (HT). Al-10 wt%Si coating is essential for improving the high-temperature corrosion resistance of 30MnB5 steel during the hot-stamping process. Before HT, the coating layer primarily consisted of Al, whereas after HT, Fe–Al-based intermetallic compounds were formed throughout the layer. The Zn in the coating layer applied using the cold-spraying method was not uniformly distributed before HT. However, during HT, the low-melting-point Zn melted and re-solidified, allowing it to combine with Fe diffusing from the substrate. Consequently, Zn–Al–Fe-based intermetallic compounds were formed on the surface of the coating layer. In the Zn-coated specimens, the current density near the corrosion potential tends to be lower than that of the Al–Si-coated specimens because Zn corrodes preferentially owing to its sacrificial anode effect, thereby protecting the underlying Al–Si-coated layer and steel.

## 1. Introduction

In recent years, automobile exhaust emission regulations have been reinforced to support environmental protection and energy efficiency in accordance with global efforts toward carbon neutrality and the reduction in greenhouse gas emissions [1,2]. In response to stricter environmental regulations, the automotive industry is moving toward electric and hydrogen-fuel-cell vehicles [3,4,5]. Nevertheless, the high costs of these technologies and the challenge of immediately replacing a vast number of internal combustion engine vehicles have delayed this transition. One of the most efficient strategies to solve this problem is to reduce the weight of automobiles [6]. Automakers are actively utilizing lightweight materials and ultra-high-strength steels to achieve this, and optimized structural design and the replacement of certain metallic components with polymers have been widely adopted as effective lightweighting solutions [7,8,9,10]. However, lightweight materials are often expensive and may not fully ensure passenger safety in the event of a vehicle collision [11,12]. In addition, some composites and natural fibers have demonstrated comparable safety performance to conventional vehicles’ materials; however, their application remains economically unfeasible [13]. Consequently, ultrahigh-strength steel, which offers excellent strength at a relatively low cost, has been adopted as a key structural material [14,15]. This type of steel is typically manufactured using a hot-stamping process. Nevertheless, several challenges arise in the application of ultrahigh-strength steels.

With increasing strength levels in ultrahigh-strength steels, high carbon contents are often required. However, this makes the steel more susceptible to high-temperature oxidation and decarburization during heat treatment (HT), which in turn leads to a reduction in corrosion resistance. To address these issues, Al–Si coatings are considered essential for hot-stamped steel sheets [16,17,18,19,20]. Although the Al-10%Si coating provides excellent resistance to high-temperature oxidation during hot stamping, its long-term corrosion resistance in harsh settings [21], such as marine environments or regions where de-icing salts are used, remains a concern. Zn-based coatings, such as galvanized steel sheets, have been developed to improve the corrosion resistance of Al–Si coatings; however, they suffer from poor heat formability [22,23]. Although Al–Si–Zn alloy coatings are used for specific purposes, they are costly. Furthermore, as additional alloying elements such as Zn, Al, Mg, and Si are incorporated, not only cost concerns but also the combined effects of these elements become significant, making optimization necessary [24,25]. In this study, a method was developed to improve the corrosion resistance of Al–Si coatings while maintaining their high-temperature formability. The most common techniques used to deposit Al–Si and Zn coatings on steel sheets include hot-dip galvanizing, electroplating, and thermal spraying [26,27]. However, these conventional methods generally require high processing temperatures or molten baths, which can lead to oxidation, alloy element evaporation, or microstructural degradation of the substrate [28]. In comparison, the cold spray process offers a solid-state deposition route that operates at relatively low temperatures, thereby minimizing oxidation and preserving the original properties of both the feedstock and the substrate. Moreover, cold-sprayed coatings typically exhibit high density, strong adhesion, and low residual stress without thermal distortion, making this process an attractive alternative for corrosion-resistant Al–Si and Zn coatings [29,30]. However, during hot stamping process, Zn-coated steels are exposed to temperatures above the melting point of Zn (~419 °C). At high temperatures, Zn can exist in a liquid state and, under applied stress, infiltrate along grain boundaries of the steel substrate. This infiltration reduces ductility and promotes crack initiation, leading to liquid metal embrittlement (LME). Therefore, after forming a Zn coating layer on the Al–Si coating layer via cold spraying, the corrosion resistances of the Al–Si and Al–Si + Zn coatings before and after HT were compared. Furthermore, the practicality of the developed technology was enhanced by determining the mechanism responsible for the improved corrosion resistance imparted by the additional Zn coating layer. The main contributions of this study are as follows: (i) elucidation of the phase transformation behavior and corrosion mechanism of Al–Si-based coating layers under hot stamping conditions; (ii) proposal of a practical process alternative through the introduction of a cold-sprayed topmost Zn coating layer; and (iii) provision of performance validation for industrial applicability and design guidelines for materials and processes via a simple and cost-effective coating method.

## 2. Materials and Methods

**Figure 1:**
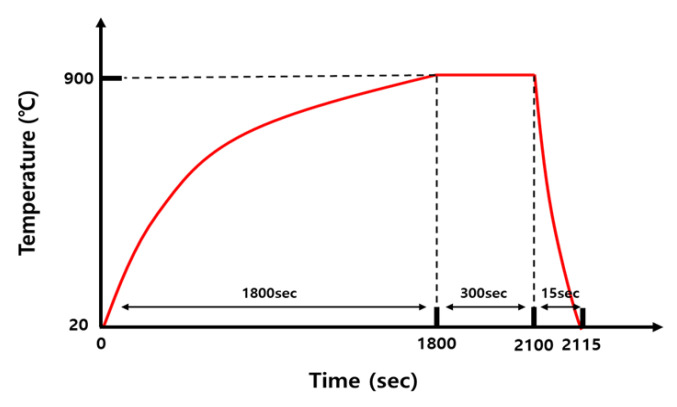
Schematic of heat-treatment cycles.

The base metal (BM) used in this study was a 30MnB5 hot-stamping steel sheet with a thickness of 1.2 mm. The chemical compositions listed in Table 1 were determined using electron probe micro-analyzer (EPMA) on a JXA-8530F instrument (JEOL Ltd., Akishima, Tokyo, Japan), following the ASTM E1508-12a standard [31]. Accordingly, in order to simulate the actual hot-stamping process, the sample was heated from 20 °C to 900 °C over ~30 min in a vertical electric furnace (3S ENG Co., Ltd., Busan, Republic of Korea), following the heat-treatment cycle shown in Figure 1. This was followed by holding at 900 °C for 5 min and water cooling for 15 s.

**Figure 2:**
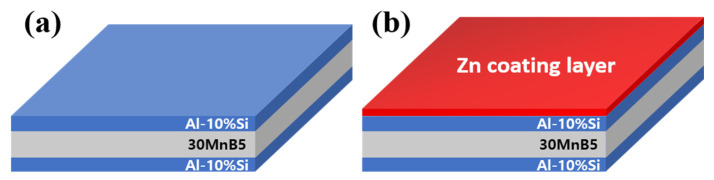
Diagram of the coating-layer shape of each specimen: (**a**) Al–Si coating, and (**b**) Al–Si + Zn coating layers.

Figure 2 schematically shows the cross-sectional structures of the specimens: (a) with Al-10 wt%Si coating on both sides of the 30MnB5 BM and (b) with the same coating and a cold-sprayed Zn layer on one side. Cold spraying was performed using a high-pressure cold spray system, and a high-purity Zn powder (>99.9%) with an average particle size of 10–60 μm was used as the feedstock material. Nitrogen gas at a pressure of 3 MPa and a temperature of 300 °C served as the propellant. In addition, the nozzle traverse speed was maintained at 500 mm/s, with a stand-off distance of 20 mm.

Optical microscopy (OM: SOK Co., Ltd., Seoul, Republic of Korea) was used to observe the microstructure of the coating layer, intermetallic compound (IMC) layer, and BM of 30MnB5 before and after HT, as well as to confirm the coating morphology and measure its thickness. Scanning electron microscopy (TESCAN ORSAY HOLDING, Brno, Czech Republic)–energy dispersive X-ray spectroscopy mapping was employed to analyze the elemental distributions of the Al–Si coating layer and BM before and after HT. Additionally, point analyses were performed on the coating, IMC, and BM regions, focusing on Fe, Al, and Si to evaluate the compositional changes across the layers. Electron backscatter diffraction was used to investigate the phase-transformation behavior in the coating layer and 30MnB5 BM of the Al–Si-coated specimens before and after HT. In addition, electron probe microanalysis mapping was used to characterize the thin powdered zinc coating on the surface, and specimens with and without the coating were compared before and after HT. To measure the hardness distributions of the coating layer, IMC, and BM in each specimen before and after heat treatment, a very light load of 5 gf was applied, with a dwell time of 10 s, and the indentation interval was set to 20 μm. To accurately assess the hardness distribution of each layer, 20 measurements were performed for the coating layer, IMC, and BM.

Electrochemical tests were performed using the specimen as the working electrode, platinum as the counter electrode, and Ag/AgCl (3.5 M KCl) as the reference electrode. To effectively evaluate the corrosion behavior of hot-stamped steel sheets with zinc coatings while maintaining experimental stability, a 2 wt% NaCl aqueous solution was used as the electrolyte, and dissolved oxygen was removed prior to measurement. The solution temperature was controlled at 27 °C in a temperature–humidity chamber, with a 500 s equilibration period applied to stabilize the system. Potentiodynamic polarization was then conducted from −1.3 V to 0.2 V at a scan rate of 0.167 mV/s to allow sufficient electrochemical reactions at each potential. These electrochemical tests were conducted according to the ASTM G5-14 standard, with the experimental setup, electrode configuration, and other parameters selected in accordance with this guideline [32].

## 3. Results and Discussions

### 3.1. Microstructural and Compositional Behavior of 30MnB5 Steel Before/After Heat Treatment

**Figure 3:**
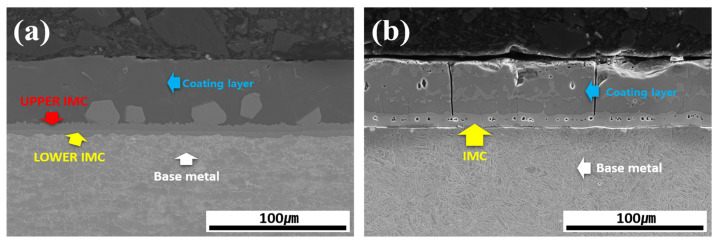
Cross-sectional morphology of the Al–Si-coated 30MnB5 BM specimen: (**a**) before, and (**b**) after HT.

Figure 3 shows the cross-sectional morphology of the Al–Si-coated 30MnB5 BM specimen before and after HT. Prior to HT, the cross-section consisted of four layers: coating layer, upper IMC, lower IMC, and BM. The coating layer displayed an Al–Si coating morphology with a smooth surface and uniform thickness. After HT, the coating layer exhibited an uneven surface and variable thickness accompanied by the formation of new compounds within the coating layer. Furthermore, the previously distinct upper and lower IMC layers merged into a single IMC layer, resulting in a three-layer structure composed of a coating layer, IMC, and BM.

**Figure 4:**
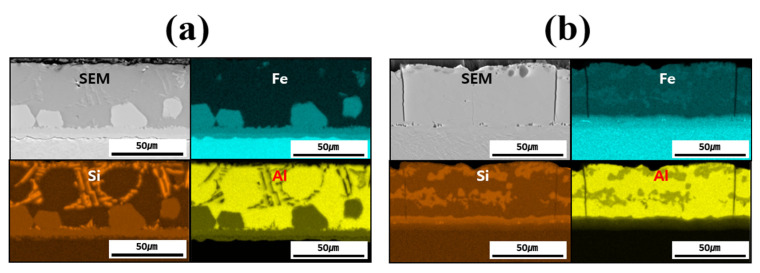
EDS-based elemental maps of the coating layer, IMC, and BM for each specimen: (**a**) before, and (**b**) after HT.

Figure 4 shows the EDS-based elemental maps used to confirm the compositional distribution of each layer (coating layer, IMC, and BM) before and after heat treating each specimen. The main elements analyzed were Fe from the BM and Al and Si from the coating layer. A comparison of the results before and after HT confirmed that Fe diffused from the BM into the coating layer during HT. In addition, while Si was confined to the IMC and parts of the coating layer before HT, it was uniformly distributed throughout the coating layer and IMC after HT. Al was found to bond with Fe only in the vicinity of the IMC before HT; after HT, Al–Fe bonding occurred across the entire coating layer. The component distribution in each layer of the specimen was found to change owing to HT, which was also expected to influence the microstructure and mechanical properties.

**Figure 5:**
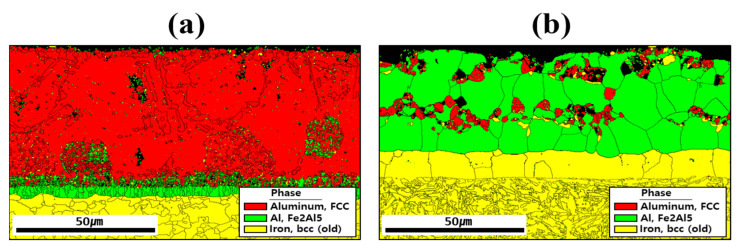
Crystal structures of the coating layer, IMC, and BM revealed by EBSD phase maps for each specimen: (**a**) before, and (**b**) after HT.

**Figure 6:**
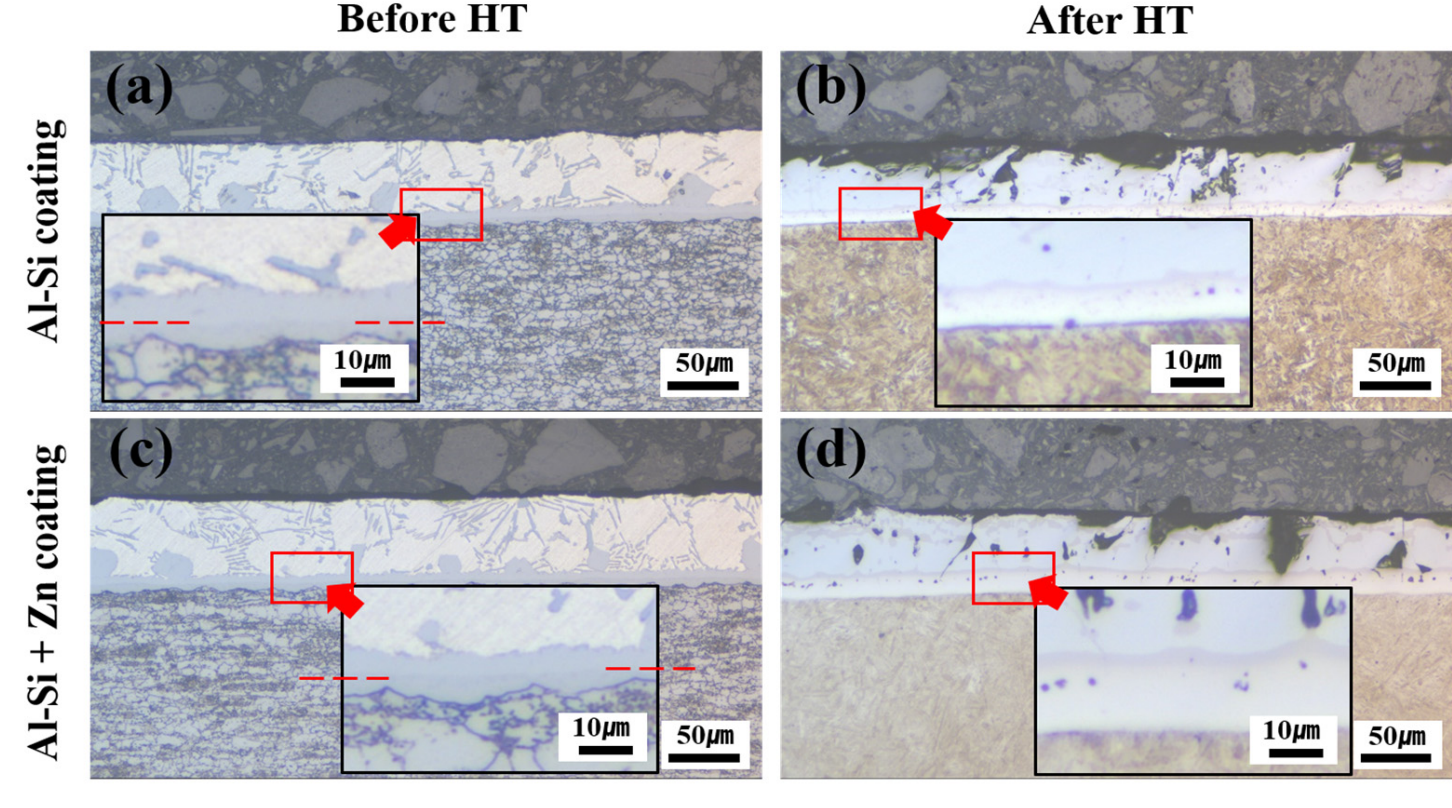
Morphologies of the coating layer, IMC, and BM in each specimen: (**a**) Al–Si-coated specimen before HT, (**b**) Al–Si-coated specimen after HT, (**c**) Al–Si + Zn-coated specimen before HT, and (**d**) Al–Si + Zn coated specimen after HT.

Figure 5 shows the EBSD phase maps used to analyze the crystal structures of the coating layer, IMC, and BM before and after HT. The results indicated that compounds formed within the coating layer, and the primary phases were aluminum (face-centered cubic: FCC), Fe_2_Al_5_ (orthorhombic), and iron (body-centered cubic: BCC) near the coating layer. Before HT, most of the Al and Si are segregated within the coating, and Si is relatively depleted in the regions where Al is predominantly segregated as shown in Figure 4a [33]. Therefore, in the regions where Al is mainly segregated, aluminum with an FCC structure is observed, whereas in the regions where Si is mainly segregated, it appears that Si has precipitated from the aluminum matrix. The division into upper and lower IMC layers shown in Figure 3a is attributed to the degree of Fe dilution, as illustrated in Figure 4a. This distinction is also evident in Figure 5a, where the IMC layer is differentiated according to the extent of Al combining with Fe to form Fe_2_Al_5_ [34]. In addition, the BM is composed of BCC-structured Fe. After HT, Fe from the BM diffused into the coating layer, and Fe_2_Al_5_ became the dominant phase throughout the layer. During this process, the proportion of FCC aluminum decreased, indicating that HT altered both the chemical composition and microstructure of the coating layer. In the BM of Figure 6b, the microstructure appears to have been refined by heat treatment and rapid cooling, which can be attributed to a phase transformation from ferrite to martensite [34,35].

Overall, HT promoted elemental diffusion between the Al and Si coating layer and the BM, leading to the formation of intermetallic compounds and modifications in the layer structure. The phase-transformation behavior of the Al–Si-coated 30MnB5 specimen after HT was consistent with the EDS-based elemental mapping results described above.

### 3.2. Microstructural and Compositional Behavior of 30MnB5 Steel Depending on the Addition of Zn Coating and Application of Heat Treatment

Figure 6 shows the morphologies of the coating layer, IMC, and BM in each specimen before and after Zn coating and HT. In the Al–Si-coated specimen before HT, the IMC layer consisted of upper and lower layers, whereas after the HT, it transformed into a single IMC layer. In the Zn powder–coated specimen (Al–Si + Zn), the coating layer exhibited a uniform thickness prior to HT. Similar to the Al–Si-coated specimens, the IMC layer was observed as separate upper and lower layers. Following HT, the Al–Si + Zn coating layer exhibited a nonuniform morphology, whereas the IMC layer was integrated into a single continuous layer.

To compare the coating layer thickness among the specimens, the coating layer, upper IMC, and lower IMC were measured prior to HT. After HT, the average thicknesses of the coating layer and IMC were obtained from 20 measurements, as presented in Table 2. Following HT, both the Al–Si-coated and Al–Si + Zn-coated specimens exhibited non-uniform coating layers, and the IMC layer was reconstructed into a single layer. Nevertheless, a distinct difference was observed in the average thickness reduction. The Al–Si-coated specimen experienced a total reduction of 8.911 μm, while the Al–Si + Zn coated specimen exhibited a smaller reduction of 4.811 μm, indicating that the average coating layer thickness reduction was less pronounced in the Al–Si + Zn coated specimen.

**Figure 7:**
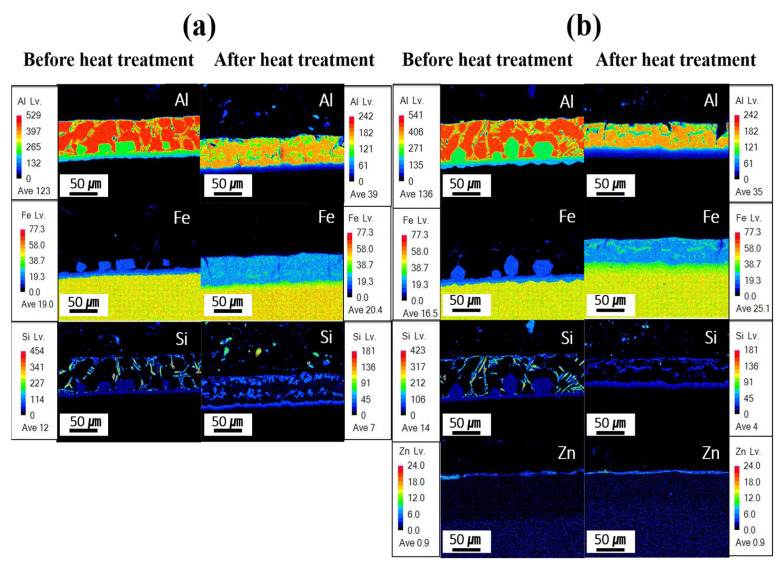
EPMA-based mapping analysis for each specimen before and after HT: (**a**) Al–Si coating, (**b**) Al–Si + Zn coating layers.

Figure 7 presents the EPMA mapping analysis results for the component segregation behavior of each specimen before and after Zn coating and HT. In the Al–Si-coated specimen before HT, the coating layer was primarily composed of Al and Si, with minimal Fe. Between the upper and lower IMC layers, Al remained the dominant element, whereas the Fe content increased in the lower IMC layer, near the BM. The BM is predominantly composed of iron. Following HT, Fe was identified in the coating layer with an Fe-to-Al atomic ratio of ~1:2, indicating the potential formation of Fe–Al–Si compounds, as presented in Table 3. In the IMC layer, a reduction in the Al content and a pronounced increase in the Fe content were observed relative to those before HT. These results indicate that Fe diffuses from the BM into the coating layer during HT. Before HT, the Zn powder–coated specimen exhibited a component behavior comparable to that of the Al–Si-coated specimens without HT. At this stage, a Zn powder coating layer formed in an uneven distribution on the Al–Si coating surface. As Zn powder was coated through the cold-spray process, an uneven Zn coating layer was formed due to the presence of uncoated areas and partial peeling of the coating. After HT, the Al–Si + Zn-coated specimen exhibited component behavior nearly identical to that of the heat-treated Al–Si-coated specimens, although the Zn-coated layer was more uniformly distributed compared with its state before HT. These results indicate that during heat treatment, Zn, which has a low melting point, melts and subsequently re-solidifies. This process, combined with the Fe diffused into the coating layer, can lead to the formation of a Zn–Al–Fe intermetallic compound on the coating surface. This confirms that Zn formed a uniform coating layer after heat treatment. As shown in Figure 7b, the Zn-based coating layer is very thin (less than 5 μm) and could therefore only be identified through compositional analysis. To verify the reproducibility of the Zn coating properties, three specimens were examined before and after heat treatment, and consistent trends were observed. Moreover, the Zn distribution of the coating layer and BM after HT is attributed to the electron-scattering effect during EPMA analysis, indicating that Zn does not truly diffuse into the BM.

**Figure 8:**
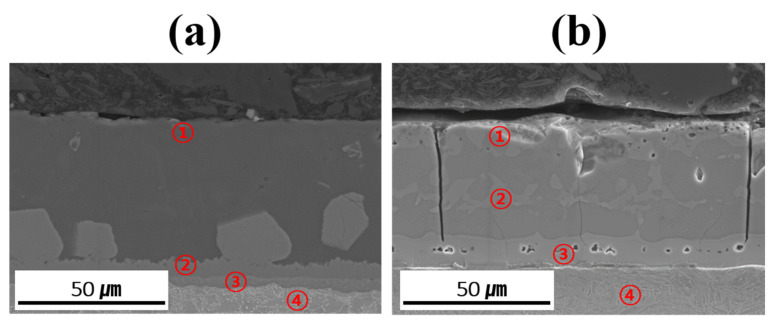
Measurement location for point analysis of each coated specimen before and after HT: (**a**) Al–Si coating, (**b**) Al–Si + Zn coating layers.

Table 3 summarizes the chemical compositions of the coating layer, IMC, and BM in each specimen before and after Zn coating and HT. The relative concentration trends of Al, Si, and Fe in each layer were determined using EDS point analysis to verify and support the findings of the EPMA mapping analysis. However, owing to the limitations in absolute quantification, the EDS data were only used to roughly compare the elemental distributions in the coating layer, IMC, and BM. The measurement locations provided in Table 3 are indicated by red numbers in Figure 8. The coating layer of each specimen was analyzed near its upper surface to confirm the presence of Zn. Before HT, the IMC layer was divided into upper and lower parts, whereas in the heat-treated specimens, Fe–Al–Si compounds were formed within the coating layer; thus, two regions were analyzed in each case. Evaluating the component behavior of each specimen (Al–Si-and Al–Si–Zn-coated) before and after HT revealed that the chemical compositions of each region (coating layer, IMC, and BM) were consistent with the component segregation observed in the previous EPMA analysis.

### 3.3. Mechanical Properties of 30MnB5 Steel Depending on the Addition of Zn Coating and Application of Heat Treatment

**Figure 9:**
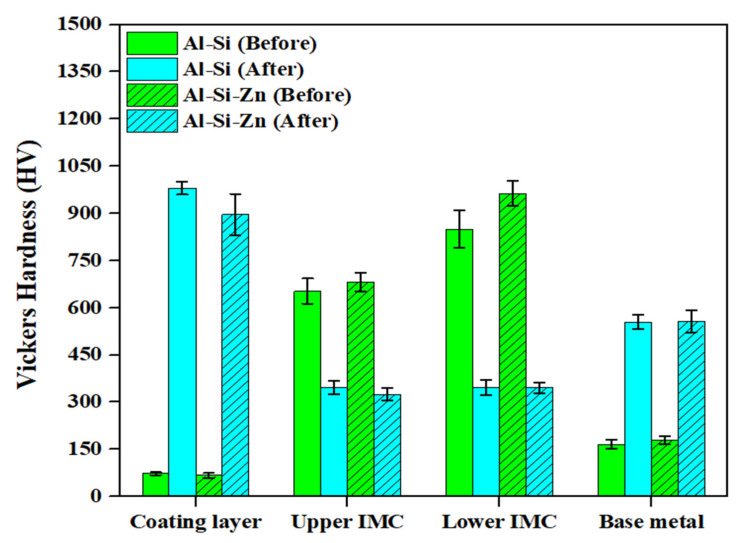
Average hardness values of the coating layer, IMC, and BM in Al–Si-coated and Al–Si + Zn-coated specimen before and after HT.

Figure 9 shows the hardness distributions of the coating layer, IMC, and BM in each specimen before and after Zn coating and HT. The Vickers hardness values represent the average values obtained from 20 measurements for each layer of the specimens. Before HT, the Al–Si and Al–Si + Zn coating layers exhibited low average hardness values of 73.5 HV and 67.4 HV, respectively, which can be attributed to the high aluminum content in the coating composition (90 wt% Al, 10 wt% Si). The significant increase in coating layer hardness after HT, to 980.5 HV and 896.4 HV, respectively, is attributed to the diffusion of Fe from the BM into the coating layer during HT, leading to the formation of IMCs. Although the average hardness of the Al–Si and Al–Si + Zn coating layers after HT differed by ~90 HV, this deviation indicates that this difference was not significant. Before HT, the IMC layer exhibited a high hardness of 600–900 HV, which decreased to ~300 HV after HT. According to the EBSD results in Figure 5, the decrease in hardness observed in the region expected to be the IMC layer after heat treatment is attributed to the dilution of Fe from the BM into the IMC layer. This leads to the phase transformation of Fe_2_Al_5_ into ferrite, which has the same BCC structure as the BM, or into a Fe-rich IMC. The average hardness of the IMC layer in all the specimens differed from that of the BM after HT, likely because residual Al and Si were retained in the IMC layer, as confirmed by the EDS results. The hardness of the BM before HT was relatively low (~170 HV) owing to its ferritic BCC structure at room temperature. After HT, the hardness increased markedly to ~550 HV, which can be attributed to the phase transformation of ferrite (BCC) into austenite (γ-Fe) during heating, followed by the formation of martensite with a BCT structure during rapid cooling [36,37]. The hardness distribution results for each layer before and after HT clearly demonstrate the impact of the microstructural behavior on the hardness.

### 3.4. Corrosion Behavior of 30MnB5 Steel Depending on the Addition of Zn Coating and Application of Heat Treatment

**Figure 10:**
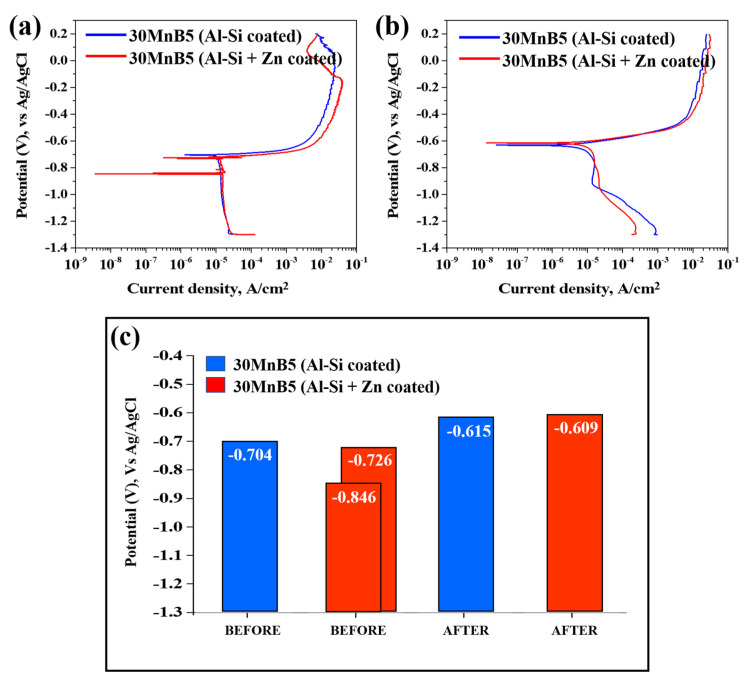
Polarization test results illustrating the corrosion behavior of each specimen: (**a**) before, (**b**) after HT, and (**c**) bar graph of corrosion potential values.

**Figure 11:**
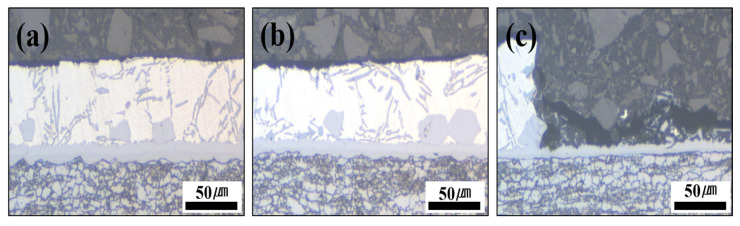
Cross-section photographs of the Al–Si + Zn-coated specimen by interrupting the polarization at three potential stages: (**a**) −800 mV, (**b**) −650 mV, and (**c**) −500 mV.

Figure 10 presents the polarization test results illustrating the corrosion behavior of each specimen before and after Zn coating and HT. The graph on the right illustrates the corrosion potentials of each specimen before and after HT. Prior to comparing and analyzing the kinetic polarization results, the distinctive features of the Zn-coated specimen before HT are described. As shown in Figure 10, unlike the other cases, the polarization curve of the Zn-coated specimen before HT exhibits two corrosion potentials (E_corr_) at −846 mV and −725 mV. As the repeated experiments produced consistent results, the test was performed under identical conditions, and the cross-sections were observed by interrupting the polarization at three potential stages (−800, −650, and −500 mV). The cross-sections were examined using OM at ×200 magnification and as shown in Figure 11.

The cross-sectional analyses of the specimens interrupted at −800 mV and −650 mV showed that no damage occurred in the Al–Si coating layer at −800 mV, and only minor surface changes were observed at −650 mV compared with the pre-test coating. However, when the potential scan entered the anodic region (−500 mV), the cross-section revealed that the Al–Si coating was locally and completely removed in the area exposed to the NaCl solution, while some regions remained intact. The residual layer adjacent to the BM in the corroded area was identified as the Fe–Al IMC (Figure 10). Although no damage was detected in the Al–Si coating at −800 mV, a distinct peak appeared at −846 mV, which can be attributed to the preferential dissolution of the surface Zn layer before HT due to its sacrificial anode effect. Consequently, the Al–Si coating remained undamaged. These observations indicate that corrosion of the Al–Si coating layer initiates only after the potential shifts into the anodic region.

Figure 7b confirms that the Zn coating exhibited an uneven morphology prior to HT, and that both the Zn and Al–Si coating layers were simultaneously exposed when subjected to NaCl solution. Prior to the experiment, the solution and cell temperatures were controlled, and a 500 s stabilization period was implemented to ensure experimental reproducibility, during which a thin oxide layer developed on the specimen surface. Al–Si-coated specimens are expected to form Al_2_O_3_, and Al–Si + Zn-coated specimens are anticipated to form both ZnO and Al_2_O_3_. Overall, as illustrated in Figure 10a, the two corrosion potentials observed in the Al–Si + Zn coating layer represent distinct electrochemical stages. The E_corr_ is the mixed potential at which the anodic and cathodic reactions are balanced. The potential at −846 mV corresponds to the sacrificial anode behavior of Zn, while the potential at −726 mV indicates the activation of the underlying Al–Si layer following Zn depletion. When the potential shifts positively beyond E_corr_, anodic dissolution becomes dominant, initiating corrosion of the Al–Si coating.

Figure 10b shows that the corrosion potential of the Al–Si + Zn-coated specimen decreased in one potential step following HT. Both specimens demonstrated an increasing trend in corrosion potential up to −0.6 V. During the heat-treatment process, Fe from the substrate diffused into the Al–Si coating, forming an Fe–Al–Si intermetallic compound. The ~100 mV increase in corrosion potential following HT is primarily attributed to microstructural changes within the coating, such as Fe–Al interdiffusion and the formation of Fe_2_Al_5_ intermetallic compounds, which alter the overall electrochemical activity of the surface. These reactions also promote the formation of a mixed oxide layer composed mainly of Al_2_O_3_, ZnO, and Fe_2_O_3_, which further stabilizes the surface [38,39]. Notably, the oxide layers in the Al–Si + Zn-coated specimen likely developed more extensively than those in the Al–Si-coated specimen owing to the 500 s stabilization period prior to the experiment.

At approximately −0.6 V, the surface oxide layers on the coating become unstable and begin to break down, thereby accelerating the corrosion process. The lower current density observed for the Al–Si + Zn-coated specimen at this potential can be attributed to the sacrificial anode behavior of Zn, consistent with that observed before HT. Zn preferentially dissolves, forming a thin and continuous corrosion product layer that temporarily suppresses the ingress of chlorides. As the potential shifts further in the anodic direction, this protective layer is eventually destroyed, exposing the IMC layer and promoting electrochemical reactions, which lead to a rapid increase in current density.

In summary, the sacrificial anode effect of Zn was observed both before and after HT, and the corrosion products formed during rapid Zn corrosion acted as physical barriers, delaying the corrosion rate. Therefore, applying a Zn powder coating in addition to an Al-10%Si coating on automotive hot-stamping steel (30MnB5) can effectively slow corrosion through this sacrificial anode mechanism.

## 4. Conclusions

In this study, Al-10%Si coating was applied to 30MnB5 hot-stamped steel, followed by the addition of a Zn powder coating. The investigation focused on the influence of the Zn coating on the microstructural evolution and corrosion behavior, leading to the following conclusions:

(1) Before HT, the Al–Si coating mainly consisted of Al, whereas after HT, Fe diffusion from the BM resulted in the formation of Fe–Al–Si intermetallic compounds throughout the layer.

(2) Before HT, the cold-sprayed Zn coating was unevenly distributed. During HT, Zn melts and re-solidifies, reacting with diffused Fe to form Zn–Al–Fe intermetallic compounds, resulting in a uniform Zn coating layer.

(3) Before HT, the coating and base material exhibited low hardness, whereas the IMC exhibited high hardness. After HT, the coating and base material hardened owing to Fe diffusion and martensitic transformation, whereas the IMC softened. The formation of Fe_2_Al_5_ in the coating promotes Al_2_O_3_ oxide layer formation, enhancing corrosion resistance.

(4) HT shifted the corrosion potential from −0.7 V to −0.6 V owing to Fe–Al compound formation and Al_2_O_3_ film development. Zn-coated specimens exhibited lower current densities near the corrosion potential owing to the sacrificial anode effect of Zn, but above the corrosion potential, their current densities increased as Zn corrosion products reacted with Cl^−^ ions.

(5) This study developed a coating technology for hot-stamped steel that employs a powdered zinc coating to protect the Al–Si coating layer and substrate through the sacrificial anode effect, thereby enhancing corrosion resistance, while rapid cooling during the heat treatment process, increases strength and contributes to weight reduction.

## Figures and Tables

**Table 1:** Chemical composition of hot-stamping boron steel 30MnB5.

Material	Chemical Composition of Base Metal (wt%)									
30MnB5 (1.2 mm)	Al	B	C	Cr	Mn	N	Ni	Si	Ti	Fe
	0.03	0.0034	0.31	0.21	1.31	0.005	0.038	0.24	0.036	Bal.

**Table 2:** Measurement results of coating layer length for each specimen before and after HT: (**a**) Al–Si and (**b**) Al–Si + Zn coating layers.

Each Layer Thickness (µm)	(a)		(b)	
	Before	After	Before	After
**Coating layer**	13.042 ± 0.738	11.106 ± 1.278	9.744 ± 0.778	7.674 ± 0.917
**Upper IMC**	9.546 ± 0.797	8.284 ± 0.916	16.403 ± 1.247	14.924 ± 1.231
**Lower IMC**	36.596 ± 1.681	30.883 ± 2.240	40.470 ± 1.889	39.208 ± 1.980
**Total length**	59.184 ± 1.887	50.273 ± 2.662	66.617 ± 1.898	61.806 ± 1.987

**Table 3:** EDS-based point-analysis results for each coated specimen before and after HT: (**a**) Al–Si and (**b**) Al–Si + Zn coated specimens.

Measurement location			Al	Si	Fe	Zn	Others
**(a)**	**Before HT**	**①**	87.82	11.75	0.43	0	0
		**②**	67.93	12.26	19.81	0	0
		**③**	63.73	6.26	30.01	0	0
		**④**	0	0	96.32	0	3.68
	**After HT**	**①**	63.07	3.11	30.47	0	0
		**②**	59.71	6.49	33.80	0	0
		**③**	18.54	6.00	74.58	0	0.88
		**④**	0	0	96.93	0	3.07
**(b)**	**Before HT**	**①**	92.14	7.81	0	0.05	0
		**②**	66.67	13.47	19.86	0	0
		**③**	62.98	7.50	29.52	0	0
		**④**	0	0	99.14	0	0.86
	**After HT**	**①**	64.52	4.51	30.68	0.24	0
		**②**	53.99	13.12	32.89	0	0
		**③**	18.30	8.81	70.71	0	2.18
		**④**	0	0	97.71	0	2.29

## Data Availability

The original contributions presented in this study are included in the article. Further inquiries can be directed to the corresponding author.
